# Integrating multimedia models to assess nitrogen losses from the Mississippi River basin to the Gulf of Mexico

**DOI:** 10.5194/bg-15-7059-2018

**Published:** 2018

**Authors:** Yongping Yuan, Ruoyu Wang, Ellen Cooter, Limei Ran, Prasad Daggupati, Dongmei Yang, Raghavan Srinivasan, Anna Jalowska

**Affiliations:** 1 US Environmental Protection Agency, Office of Research and Development, Research Triangle Park, North Carolina, USA; 2 Department of Land, Air and Water Resources, University of California, Davis, California, USA; 3 Water Resources Engineering, University of Guelph, Guelph, Ontario, Canada; 4 Institute for the Environment, University of North Carolina at Chapel Hill, Chapel Hill, North Carolina, USA; 5 Department of Ecosystem Sciences and Management, Texas A & M, College Station, Texas, USA; 6 Department of Biological and Agricultural Engineering, Texas A & M, College Station, Texas, USA

## Abstract

This study describes and implements an integrated, multimedia, process-based system-level approach to estimating nitrogen (N) fate and transport in large river basins. The modeling system includes the following components: (1) Community Multiscale Air Quality (CMAQ),(2) Weather Research and Forecasting Model (WRF), (3) Environmental Policy Integrated Climate (EPIC), and (4) Soil and Water Assessment Tool (SWAT). The previously developed Fertilizer Emission Scenario Tool for CMAQ (FEST-C), an advanced user interface, integrated EPIC with the WRF model and CMAQ. The FEST-C system, driven by process-based WRF weather simulations, includes atmospheric N additions to agricultural cropland and agricultural cropland contributions to ammonia emissions. This study focuses on integrating the watershed hydrology and water quality model with FEST-C system so that a full multimedia assessment on water quality in large river basins to address impacts of fertilization, meteorology, and atmospheric N deposition on water quality can be achieved. Objectives of this paper are to describe how to expand the previous effort by integrating the SWAT model with the FEST-C (CMAQ/WRF/EPIC) modeling system, as well as to demonstrate application of the Integrated Modeling System (IMS) to the Mississippi River basin (MRB) to simulate streamflow and dissolved N loadings to the Gulf of Mexico (GOM). IMS simulation results generally agree with US Geological Survey (USGS) observations/estimations; the annual simulated streamflow is 218.9 mm and USGS observation is 211.1 mm and the annual simulated dissolved N is 2.1 kg ha^−1^ and the USGS estimation is 2.8 kg ha^−1^. Integrating SWAT with the CMAQ/WRF/EPIC modeling system allows for its use within large river basins without losing EPIC’s more detailed biogeochemistry processes, which will strengthen the assessment of impacts of future climate scenarios, regulatory and voluntary programs for N oxide air emissions, and land use and land management on N transport and transformation in large river basins.

## 1 Introduction

Increased nitrogen (N) fluxes from the Mississippi River basin (MRB) have been linked to increased occurrences of seasonal hypoxia in the northern Gulf of Mexico (GOM) (NSTC, 2000; USEPA, 2014; Alexander et al., 2008; Rabalais et al., 2001). Hypoxia is an environmental phenomenon in which concentration of dissolved oxygen in the water column decreases to a level that can no longer support living aquatic organisms, which, in turn, depletes valuable fisheries and disrupts ecosystems. Modeling studies have been conducted to improve understanding of factors and sources contributing to increased N export from the MRB (Alexander et al., 2008; David et al., 2010; Donner and Kucharik, 2008; Donner and Scavia, 2007; Mayorga et al., 2010; McCrackin et al., 2014; NSTC, 2000; Santhi et al., 2014). Those focused on interactions of land and water and the NSTC (2000) concluded that N loading to the GOM is related to runoff, agricultural activity, and human population densities. The current strategy of the Hypoxia Task Force (HTF), a collaborative effort of federal and state agencies, as well as tribes, is to reduce both N and phosphorous (P) losses through state-level nutrient reduction strategies and by targeting actions within watersheds where they will be most effective. There is an interim target of reducing N and P loading by 20 % (relative to the 1980–1996 baseline period) by 2025 and a goal of reducing the summer hypoxic zone to less than 5000 km^2^ by 2035 (USEPA, 2014).

However, it is not clear how atmospheric N deposition contributes to the total N load and its impact on rivers, lakes and estuaries in the MRB, particularly impacts of Clean Air Act (CAA) regulations on abatement. Furthermore, the climate is changing: temperatures are rising, snow and rainfall patterns are shifting, and more extreme climate events such as heavy rainstorms and record high temperatures are happening (USEPA, 2016). Considering the expected changes in climate during N assessment is also critical for the MRB (Donner and Scavia, 2007), because future climate scenarios may impact streamflow generation and, thus, N loads from the watershed. Finally, due to the complex N cycle and its dynamics from the atmosphere to the biosphere, through dry deposition of gaseous N species and wet deposition of dissolved N species in precipitation, the USEPA Science Advisory Board (USEPA, 2011) and the European Nitrogen Assessment (Sutton et al., 2011) emphasized the need for integrated, multimedia, and transdisciplinary approaches to evaluate N fate and transport comprehensively. Therefore, an Integrated Modeling System (IMS) linking air, land surface, and stream processes is needed to fill the research gap for integrated, multimedia modeling for N studies in large river basins.

The Community Multiscale Air Quality (CMAQ) modeling system has been developed by the USEPA for conducting air quality simulations (https://www.epa.gov/cmaq, last access: 15 November 2018), while the Weather Research and Forecasting Model (WRF) is a community next-generation mesoscale numerical weather prediction system designed for atmospheric research and forecasting applications by the US National Center for Atmospheric Research (https://www.mmm.ucar.edu/weather-research-and-forecasting-model, last access: 15 November 2018). The combined meteorology and air quality modeling system (WRF/CMAQ) is an important decision support tool that is used to help understand the chemical and physical processes for research and policymaking to mitigate harmful effects of air pollution on human health and the environment around the world (Cohan et al., 2007; Wang et al., 2010; Compton et al., 2011). This system (WRF/CMAQ) has long been used by the federal and state governments and institutions in the United States and around the world for air quality research and regulatory decisions.

**Figure 1.**
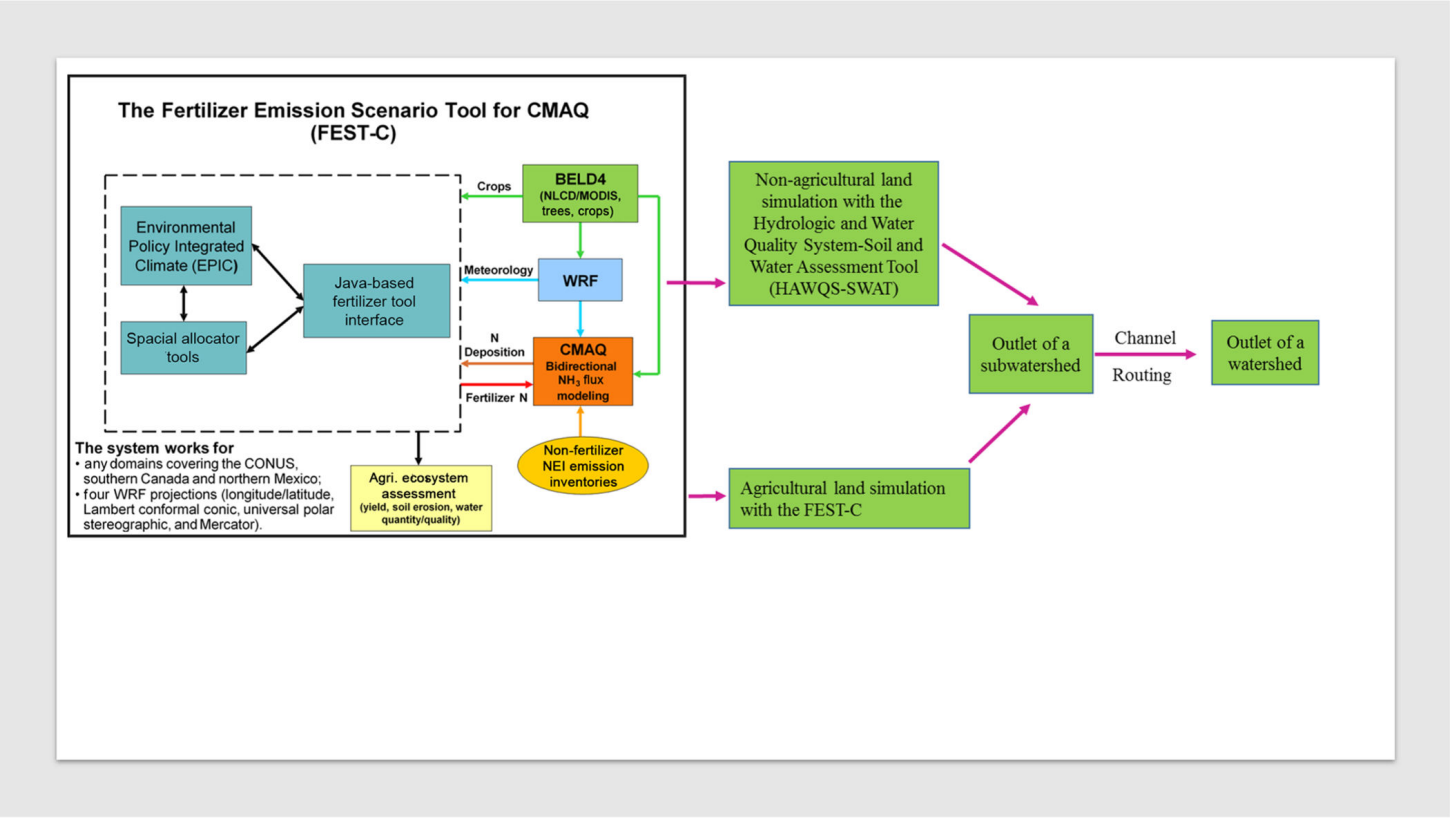
Integration of FEST-C (EPIC/WRF/CMAQ) and SWAT: EPIC was used to simulate agricultural land because of its complexity in simulating agricultural production and related pollutant loadings, as well as its interaction with CMAQ and WRF. HAWQS-SWAT simulates non-agricultural land and takes in FEST-C output from agricultural land at an outlet of a subwatershed, then simulates stream and channel processes, and routes combined loadings to the outlet.

During air quality simulations, it is often a challenge to accurately estimate NH_3_ emissions from agricultural land because N fertilization varies spatially and temporally by production types (e.g., corn vs. soybean) and locations (e.g., different soil and weather). Therefore, the USEPA developed the Fertilizer Emission Scenario Tool for CMAQ (FEST-C) system (Cooter et al., 2012; Ran et al., 2010), an advanced user interface, to integrate the Environmental Policy Integrated Climate (EPIC) model (Williams, 1995, 1990; Williams and Arnold, 1996), a field-scale agricultural biogeochemical model, with the WRF model (Skamarock et al., 2008) and CMAQ; and WRF/CMAQ simulates mesoscale meteorology and air quality (Fig. 1). The FEST-C system (EPIC/WRF/CMAQ) simulates daily fertilizer application to agricultural lands for bidirectional ammonia (NH_3_) modeling (Bash et al., 2013; Pleim et al., 2013) in the CMAQ model (Byun and Schere, 2006; Appel et al., 2017) and is useful for assessing impacts of agricultural fertilization and management practices not only on air quality (NH_3_) (Fu et al., 2015) and climate (nitrous oxide (N_2_O)) (Cooter et al., 2010), but also on crop yield, soil erosion, and hydro-ecosystems. The FEST-C system consists of field-scale models, but it provides an excellent platform for the IMS linking air, land surface, and stream processes for a full multimedia assessment on water quality. Thus, the next step toward this full multimedia assessment is to integrate the FEST-C system with water-shed processes and/or watershed hydrology and water quality models. We fill this gap by proposing to integrate the Soil and Water Assessment Tool (SWAT) with the FEST-C system to address impacts of fertilization, meteorology, and N deposition from the FEST-C modeling system on water quality.

SWAT (Arnold et al., 2012; Gassman et al., 2007; Neitsch et al., 2011) has been widely applied to evaluate best management practices, alternative land use and land management, and climate change on pollutant losses to streams within a watershed (Chaplot et al., 2004; Gassman et al., 2007; Johnson et al., 2015; Santhi et al., 2006; Vaché et al., 2002). In the past, SWAT applications focused on evaluating land use and land management and/or climate change on water quality, but none focused on an integrated modeling approach that accounted for air deposition as well as its interaction with climate and agricultural activities. SWAT can consume user-defined atmospheric deposition and wet deposition data from the National Atmospheric Deposition Program (http://nadp.sws.uiuc.edu/, last access: 15 November 2018) from 1980 to 2010, which are precipitation-weighted means (mg L^−1^) at a monthly time step (Neitsch et al., 2011;Yen et al., 2016). Neither climate nor agricultural activities interact with atmospheric deposition during a SWAT simulation. Integrating SWAT with FEST-C systems not only allows the FEST-C systems to work at large watershed scales, but also allows SWAT to take in dynamic atmospheric N deposition (bidirectional CMAQ) data so it can account for interac tion of air, climate, and agricultural activities. Furthermore, EPIC can provide a more detailed field-level biogeochemical processes simulation than SWAT. This integrated modeling system allows us to look at all potential sources of N from a watershed in a dynamic way and assess the impact of CAA amendment regulations, climate change, and land use and land management changes on N loadings in large river basins such as the MRB. This effort marks a significant step forward in a more complete systems-level framework for N assessment.

Due to the complexity of the modeling system and the scale of targeted application, our study focuses on model integration and proof of concept. The objectives were to (1) describe the integration of SWAT with FEST-C (EPIC/WRF/CMAQ) and (2) demonstrate the application of the integrated multimedia modeling system to the MRB to assess N loading.

## 2 Methodology

### 2.1 Overview of N transformation and transport

Nitrogen has the most complex nutrient cycle of all the mineral nutrients because it can exist in both dissolved forms and as gaseous NH_3_ or N_2_ (Burt et al., 1993). The N cycle and its dynamics in agricultural soils are complicated biological and chemical processes. Generally, major forms of N in soils are organic N associated with humus (active and stable in organic pool) and soluble forms of mineral N (mainly nitrate (NO^−^_3_) and ammonium (NH^+^_4_), with low concentration of nitrite (NO^−^_2_)). Nitrogen cycling and losses consist of the following processes: atmospheric N deposition; mineralization; immobilization; nitrification; denitrification; volatilization; biological N fixation from the atmosphere; decomposition of fresh residue; plant uptake; organic N transport in sediment; and nitrate and nitrite N losses in leaching, surface runoff, and lateral subsurface flow (Yuan et al., 2017). To simulate N transformation and transport, N mass balances summarizing N gains (mineralization, fixation, and fertilizer application) and losses (plant uptake, denitrification, volatilization, and immobilization) are established in simulation models. Usually, N mass balance is maintained for both the organic and inorganic pools. Potential N losses from agricultural soils may occur through nitrate and nitrite leaching to the subsurface or through surface runoff and organic N transport in sediment.

### 2.2 FEST-C system

#### 2.2.1 EPIC

EPIC is a semi-empirical biogeochemical process model that assesses the effect of wind and water erosion on crop productivity and evaluates management solutions that maximize crop production while reducing soil and nutrient losses (Williams et al., 1984, 2008). It is a daily time-step, field-scale model, and the computational “fields” can extend up to 100 ha in area. EPIC has been modified to provide a full biogeochemical characterization of agricultural systems since its original development.

EPIC simulates the complete N cycle: atmospheric N inputs; fertilizer/manure N applications; crop N uptake; nitrification (transformation of the NH^+^_4_ pool to NO^−^_3_); denitrification (conversion of NO^−^_3_ to produce N_2_ and N_2_O); ammonia volatilization (gaseous loss of NH_3_ that occurs when NH^+^_4_ is surface applied); decomposition; mineralization and immobilization; organic N transport on sediment; and nitrate-N losses in leaching, surface runoff, lateral subsurface flow, and tile flow. Mineralization is the process that breaks down organic N compounds in the soil to release NH^+^_4_, with concurrent release of carbon as CO_2_ in most cases (Vinten and Smith, 1993); the reverse process is immobilization by which NH^+^_4_ pool to NO^−^_3_ is microbially transformed into organic forms. Decomposition and mineralization of fresh organic N are controlled by a decay rate constant. Denitrification occurs only when soil moisture content is above field capacity. The fertilizer N is considered to dissolve immediately and contribute to the mineral N pool. Plant uptake of N is controlled by plant demand but limited by soil supply of the N. Organic N in each soil layer is partitioned into fresh and stable pools. The organic N loss is estimated using sediment yield, organic N on the soil surface layer, and an enrichment ratio; the soluble N loss is estimated by considering soluble N concentration changes in soil layers (Wang et al., 2012). EPIC was modified to accept time series of wet and dry atmospheric deposition of oxidized and reduced N from WRF/CMAQ through the FEST-C system (Cooter et al., 2012; Ran et al., 2010).

EPIC options include characterization of various tillage practices (e.g., conventional, reduced till, no till, and contour plowing) and engineering changes (e.g., construction of terraces and installation of tile drainage). This also includes a heat-unit-driven, above- and below-ground plant growth model; soil hydrology; and soil heat budgets for multiple soil layers of variable thickness. Simulation output frequency is user specified, ranging from daily to annual summaries of biogeochemical process rates, nutrient pools, and management activity, as well as edge-of-field runoff, sediment, and nutrients.

#### 2.2.2 WRF

The WRF model is a numerical weather prediction and atmospheric simulation system (Skamarock et al., 2008). It considers atmospheric thermodynamic properties and is applicable to horizontal spatial scales ranging from meters to thousands of kilometers. It has been used extensively for research and real-time forecasting throughout the world (http://www.wrf-model.org/index.php, last access: 15 November 2018). WRF was used to generate EPIC weather inputs including daily maximum and minimum temperature, precipitation, solar radiation, relative humidity, and wind speed.

#### 2.2.3 CMAQ

The CMAQ model is a community-based atmospheric chemistry and transport model designed to simulate photochemical (e.g., ozone), aerosol (e.g., PM_2.5_), and toxic (e.g., benzene) air pollutants (Byun and Schere, 2006; Appel et al., 2017). It simultaneously models multiple air pollutants including ozone, particulate matter, and a variety of gaseous elements (including N) to help air quality managers determine the best management scenarios for their communities, regions, and states. The tool can provide detailed information about air pollutant concentrations in a given area for any specified emission or climate scenario (http://www.epa.gov/air-research/community-multi-scale-air-quality-cmaq-modeling-system-air-quality-management, last access: 15 November 2018). Integrating CMAQ with EPIC through the FEST-C system provides a more dynamic, flexible, and spatially and temporally resolved estimate of NH_3_ emissions from application of N fertilizers to agricultural soils than previous factor-based NH_3_ inventories. Application of this integrated system produced a modified geospatial pattern of seasonal NH_3_ emissions that improved simulations of observed atmospheric particle nitrate concentrations which, in turn, provided EPIC with better atmospheric N inputs than inventories (Cooter et al., 2012).

#### 2.2.4 FEST-C and its enhancement

This research builds on existing FEST-C system and uses the bidirectional flux version of CMAQ (bidi-CMAQ), which represents the integration of EPIC and CMAQ models driven by WRF meteorology. The CMAQ version employs a compensation point approach to estimate the flux of NH_3_ (emission or deposition) from underlying soil and vegetated surfaces to air (Bash et al., 2013; Cooter et al., 2012). EPIC was modified to take daily time series of total wet oxidized N (g ha^−1^), total wet reduced N (g ha^−1^), total dry oxidized N (g ha^−1^), total dry reduced N (g ha^−1^), and total wet organic N (g ha^−1^) from WRF/CMAQ (Cooter et al., 2012).

The FEST-C system guides users through generating land use and crop data needed for EPIC (BLED4 in Fig. 1), creat ing daily weather and N deposition input from WRF/CMAQ; preparing EPIC site, soil, and management inputs (Spatial Allocator Tools in Fig. 1) for EPIC simulations; and extracting EPIC output for quality assurance. In addition, it also extracts initial soil and pH conditions and daily N information required by CMAQ bidirectional NH_3_ modeling. The Spatial

Allocator Tools connect EPIC with WRF/CMAQ (Fig. 1). Our effort in this study further enhanced the FEST-C system to generate SWAT-needed inputs from EPIC/WRF/CMAQ.

The target EPIC simulation resolution for integration with a gridded regional air quality model is 144 km^2^ (i.e., 12 km by 12 km rectangular grid-cells); land use at the start of the simulation period (2002) is used throughout. The 2002 county-level Census of Agriculture (fractional distribution of crops within the county with the total agricultural land use) was constrained by NLCD 2001 (Cooter et al., 2012) and the model was configured to simulate fertilization based on the plant demand using computed N stress level in simulation. The area of each crop land on a given 12 km by 12 km grid cell is known, but the exact location is not. EPIC produces edge-of-field outputs including runoff, sediment, and nutrients on a daily basis for each crop within a grid cell; outputs are unit loadings (kg ha^−1^).

### 2.3 Soil and Water Assessment Tool and Hydrologic and Water Quality System

SWAT simulates long-term impacts of land use and management changes on water, sediment, and agricultural chemical yields, at various temporal and spatial scales, in a water-shed (Arnold and Fohrer, 2005; Arnold et al., 1998; Gassman et al., 2007; Neitsch et al., 2011). It models the N cycle in the soil environment (in-field) and in stream water (in-stream). SWAT’s in-field N treatment is similar to that in EPIC, but it is less complex and does not have EPIC’s new additions (Cooter et al., 2012; Yuan et al., 2017). SWAT models in-stream nutrient processes using kinetic routines from QUAL2E, an in-stream water quality model (Brown and Barnwell, 1987). In-stream transformation of different N species is governed by growth and decay of algae, water temperature, biological oxidation rates for conversion of different N species, and settling of organic N with sediment. The amount of organic N in the stream may be increased by conversion of N in algae biomass to organic N and decreased by conversion of organic N to NH^+^_4_ and settling with sediment. The amount of ammonium may be increased by mineralization of organic N and diffusion of benthic ammonium N as a source and decreased by conversion of NH^+^_4_ to NO^−^_2_ or uptake of NH^+^_4_ by algae. Conversion of NO^−^_2_ to NO^−^_3_ is faster than conversion of NH^+^_4_ to NO^−^_2_; the amount of nitrite is therefore usually very small in streams. The amount of nitrate in streams can be increased by conversion of NO^−^_2_ to NO^−^_3_ and decreased by algae uptake. SWAT considers water runoff and loadings of sediment and other constituents, including point sources (e.g., sewage treatment plants), from land areas to and along the channel network and can be summarized on a daily, monthly, yearly, or average annual basis (Neitsch et al., 2011).

The Hydrologic and Water Quality System (HAWQS1.0) (https://epahawqs.tamu.edu/, last access: 15 November 2018) was recently developed by the USEPA Office of Water to enhance the usability of SWAT in simulating effects of land management practices based on an extensive array of crops, soils, natural vegetation types, land uses, and climate change scenarios on hydrology and water quality. HAWQS is a web-based, interactive water quantity and quality modeling system that employs SWAT as its core engine (Yen et al., 2016). It provides interactive web interfaces, maps, and preloaded input data including NHD Plus; land use and land management (NLCD 2006 combined with 2010 and 2011 crop data layer from the USDA National Agricultural Statistics Service, NASS, survey to differentiate agricultural land use); soil; climate; atmospheric deposition of N; and USGS data of streamflow and pollutants. Daily weather data implemented in HAWQS are from the National Oceanic and Atmospheric Administration’s National Centers for Environmental Information (NOAA-NCEI); the atmospheric deposition implemented in HAWQS is from the National Atmospheric Deposition Program which monitors precipitation chemistry (http://nadp.sws.uiuc.edu/NADP/, last access: 15 November 2018). In addition, the SWAT default parameters used by HAWQS have been preliminarily calibrated. HAWQS serves three different spatial resolutions (8-, 10-, and 12-digit hydrologic unit codes, HUCs) and varying temporal scales (daily, monthly, or annual time steps) (Yen et al., 2016).

### 2.4 Integrated Modeling System

#### 2.4.1 Integration of SWAT and EPIC for the Integrated Modeling System

EPIC was used to simulate agricultural land because of its complexity in simulating agricultural production and related pollutant loadings, as well as its interaction and compatibility with CMAQ and WRF. EPIC is a field-scale model, however, and can only simulate edge-of-field loadings from agricultural land; landscape processes from fields to reaches, channel routing, and in-stream water quality processes are not considered. Furthermore, EPIC does not simulate non-agricultural land. Therefore, SWAT was used to simulate non-agricultural land and stream processes for the Integrated Modeling System (IMS). SWAT divides a watershed into subwatersheds or sub-basins, which are further partitioned into a series of hydrological response units (HRUs), by setting a threshold percentage of dominant land use, soil type, and slope group. An HRU is assumed to be homogeneous in hydrologic response and consists of homogeneous land use and land management, soil, and slope (Gassman et al., 2007; Williams et al., 2008; Neitsch et al., 2011; Yen et al., 2016). Hydrological components, soil erosion and sediment yield, and nutrient cycles are simulated for each HRU, and yields from HRUs are aggregated for the subwatersheds. To integrate EPIC with SWAT in the IMS, loadings for all crops (agricultural land) from EPIC grids within each subwatershed or subbasin are aggregated into one value and expressed as mass (Table 1); the aggregated value is treated as a point source and directly introduced into the outlet of each subwatershed where it combines with loadings from non-agricultural land, as shown in Fig. 1. Together, loadings of runoff, sediment, and chemicals are routed from each subwatershed through a channel network to the outlet of the watershed.

This approach assumes no routing inside each subwater-shed to the pour point (i.e., no field-to-field routing). A delivery ratio (DR) method is thus used to account for the stream processes inside each subwatershed (Santhi et al., 2011; Wang et al., 2011). DR refers to the fraction of total soil and nutrient loss from fields within the subwatershed that actually reaches the nearest stream.

#### 2.4.2 Weather and atmospheric N deposition for the Integrated Modeling System

Both SWAT and EPIC require daily time series of radiation, maximum and minimum temperature, precipitation, relative humidity, and 10 m wind speed conditions. These data can come from historical observations, be simulated (e.g., data by WRF), or be a combination of both. Through the FEST-C system, EPIC receives WRF weather inputs for each 12 km by 12 km grid. WRF climate data of the 12 km by 12 km grid were aggregated to an 8-digit HUC level to run a SWAT simulation on non-agricultural land, because SWAT requires one weather file for each subbasin. Again, through the FESTC system, EPIC receives CMAQ atmospheric N deposition for each 12 km by 12 km grid (Table 2). Similarly, SWAT requires one deposition file for each 8-digit HUC; thus, the CMAQ deposition data for each 12 km by 12 km grid within each HUC8 were aggregated into one file and used for SWAT simulation on non-agricultural land (Table 2). The IMS simulation uses grid-based climate forcing by WRF because it is fully integrated with the air quality model CMAQ, which reflects N exchange between the land surface and atmosphere. Furthermore, the IMS simulation uses grid-based CMAQ atmospheric N deposition for agricultural land because it is fully integrated with the air quality model CMAQ. For non-agricultural land, both atmospheric wet deposition of ammonium (mg L^−1^) and atmospheric wet deposition of nitrate (mg L^−1^) for each subbasin were assumed to be zero. Daily total wet and dry oxidized N are summed to provide atmospheric deposition of nitrates; daily total wet and dry reduced N are summed to provide atmospheric deposition of ammonium (kg ha^−1^ day^−1^), as shown in Table 2.

### 2.5 IMS implementation on the MRB

**Figure 2.**
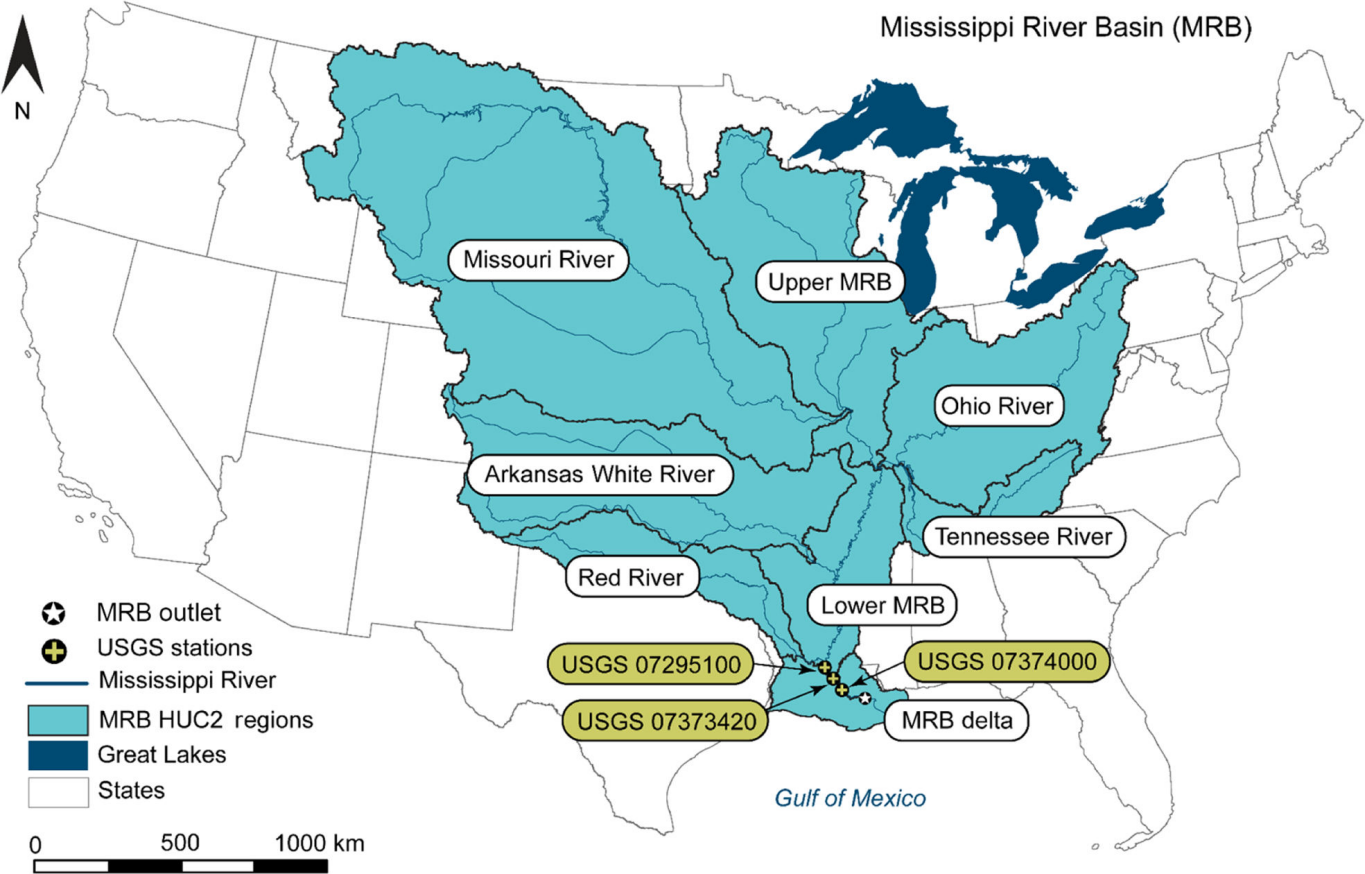
Geographic location of the Mississippi River basin (MRB): black dots with a cross indicate the USGS stations, located close to the outlet of the MRB and used to evaluate models’ performance; the black dot with a star indicates the outlet of the MRB.

The MRB including Missouri, Arkansas Red–White, Ohio–Tennessee, and upper and lower MRBs (Fig. 2) drains all or part of 31 US states (41 % of the contiguous United States). The river main stem is 3700 km in length and runs from the southern Canadian border to the GOM. The watershed provides drinking water, food, industry, and recreation for mil lions of people. The largest hypoxic zone currently affecting the United States and the second largest hypoxic zone worldwide is the northern GOM, adjacent to the Mississippi River. SWAT was set up for the MRB through HAWQS at the 8-digit HUC level, where each 8-digit HUC is treated as a subbasin.

The HAWQS-SWAT simulation comprises 821 8-digit HUCs covering an area of 3 170 000 km^2^ from northern Minnesota to Baton Rouge, LA, ending at the outlet of HUC08071000, on a daily timestep (black dot with white star in Fig. 2). Although the Mississippi River continues 100 more miles south to New Orleans, where it meets GOM, the river bifurcates after Baton Rouge and not all basins contribute directly to the Mississippi River (Fig. 2). In addition, 8-digit HUCs in Canada, which also contribute to the Mississippi River, were not included, as HAWQS was developed only for the contiguous United States. Finally, all of SWAT’s necessary data (SWAT editor tables, input files, and other associated data) were downloaded so they can be used by the SWAT editor program.

Each land use type within each 8-digit HUC was treated as one HRU: each cropland was treated as one HRU and urban land was treated as one HRU, within a given 8-digit HUC. For IMS simulation, SWAT cropland output was muted by adjusting the cropland area fraction to zero (unit loading ×area fraction = 0). EPIC loadings for all cropland (agricul-tural land) within each 8-digit HUC were aggregated into one value and introduced into the outlet of each 8-digit HUC. The IMS simulation for the MRB ends at the pour point of HUC 08070100. Since the time of travel is limited mostly to a single day within each 8-digit HUC, we assume that nutrient transformations en route to the stream are negligible. Furthermore, “area” used in Table 1 (last column) refers to the agricultural land in HAWQS-SWAT; the ratio was applied to account for the agricultural land differences between EPIC and HAWQS-SWAT, if any.

### 2.6 Model simulations for the MRB

To evaluate the IMS, the following model simulations were performed.

1. HAWQS-SWAT: all SWAT inputs including climate (daily precipitation, maximum and minimum air temperature) were directly extracted from the HAWQS system; the simulation was performed from 1999 to 2010 (weather in HAWQS 1.0 ends in 2010), with the first 3 years as a warm-up period. HAWQS-SWAT uses area-weighted NOAA-NCEI observations as climate input for each subbasin (8-digit HUC); these data are interpolated using the Thiessen polygon method to create a pseudo-station for each 8-digit HUC. Air deposition used in this simulation is from the National Atmospheric Deposition Program (http://nadp.sws.uiuc.edu/, last access: 15 November 2018).
2. HAWQS-SWAT WRF: for this simulation, climate inputs (daily precipitation, maximum and minimum air temperature) were replaced with WRF-produced daily precipitation, maximum and minimum air temperature, and solar radiation; the rest of the inputs remain the same as the above simulation (HAWQS-SWAT). This simulation was performed because the FEST-C system was driven by process-based WRF weather simulations.
3. IMS simulation: EPIC simulates agricultural land. SWAT takes in EPIC loadings, simulates non-agricultural land, and performs channel-routing processes. The IMS uses grid-based climate forcing by WRF because it is fully integrated with the air quality model CMAQ, which reflects N exchange between the land surface and atmosphere. The IMS simulation was performed for 2002 to 2010. CMAQ estimates of speciated wet and dry N deposition are used for non-agricultural land.

Results from the first and second simulation are the benchmark for the IMS evaluation. Comparing results from simulations 1 and 2 is helpful for understanding the effects WRF weather data have on a model’s results. All simulations end in Baton Rouge, LA (pour point of the HUC08071000).

### 2.7 Model evaluation

Eighty-five US Geological Survey (USGS) gauge stations across the country were used to calibrate HAWQS during its development; six were from Tennessee, eighteen from Ohio, and sixteen from the upper MRB for a total of 40 in the MRB. Since default parameters used by HAWQS have been preliminarily calibrated as documented in the HAWQS Quality Assurance Project Plan (QAPP, an unpublished EPA document), no further calibration was performed. And due to the complexities at this scale, calibration would be extremely diffi-cult and require another standalone study. Our study focuses on model integration.

Although no calibration was performed, USGS gauge stations located at the main stem of the Mississippi River and close to the outlet of the MRB were identified to support model evaluation (Table 3). The location, size of the drainage area for each USGS gauge station, and time period for available flow and N data are listed in Table 3. Three USGS stations are identified (Fig. 2 and Table 3). The USGS 07373420 Mississippi River near St. Francisville, LA, with a drainage area of 2 920 000 km^2^, is a long-term USGS National Water Quality Assessment monitoring station on the Mississippi River. Discrete water quality samples were collected, but continuous streamflow was not monitored at this station. Nutrient loads delivered to the GOM estimated by the USGS are therefore a product of the concentrations of NO^−^_3_ plus NO^−^_2_ from USGS 07373420 at St. Francisville and stream-flow from 07295100 at the Mississippi River at Tarbert Landing, MS (also US Army Corps of Engineers site 01100). More information on how the load was estimated can be found in the USGS Open-File Report 2007–1080 (USGS Streamflow and Nutrient Delivery to the Gulf of Mexico, available at http://toxics.usgs.gov/hypoxia/mississippi/flux_ests/delivery/index.html last access: 15 November 2018). Nutrient loads estimated at this site were additionally evaluated for 2011 to 2013, using in situ nitrate sensors and stream-flow data collected at the USGS 07374000 station at Baton Rouge, LA, about 60 km from this station (Pellerin et al., 2015) (Fig. 2). Pellerin et al. (2015) concluded that the measured NO^−^_3_ load with in situ nitrate sensors underestimated the load at the St. Francisville station by only 3.5 % for the entire study period. Much larger differences (5 % to 20 %) were observed at daily or monthly time steps, however. High-frequency NO^−^_3_ measurements captured the variation of the load at a daily or monthly time step and improved accuracy.

Results from all simulations were compared to available USGS data to evaluate the model’s performance. Data from all three USGS stations were used (Fig. 2 and Table 3).

## 3 Results and discussion

### 3.1 Climate forcing comparison between NOAA-NCEI and WRF

**Figure 3.**
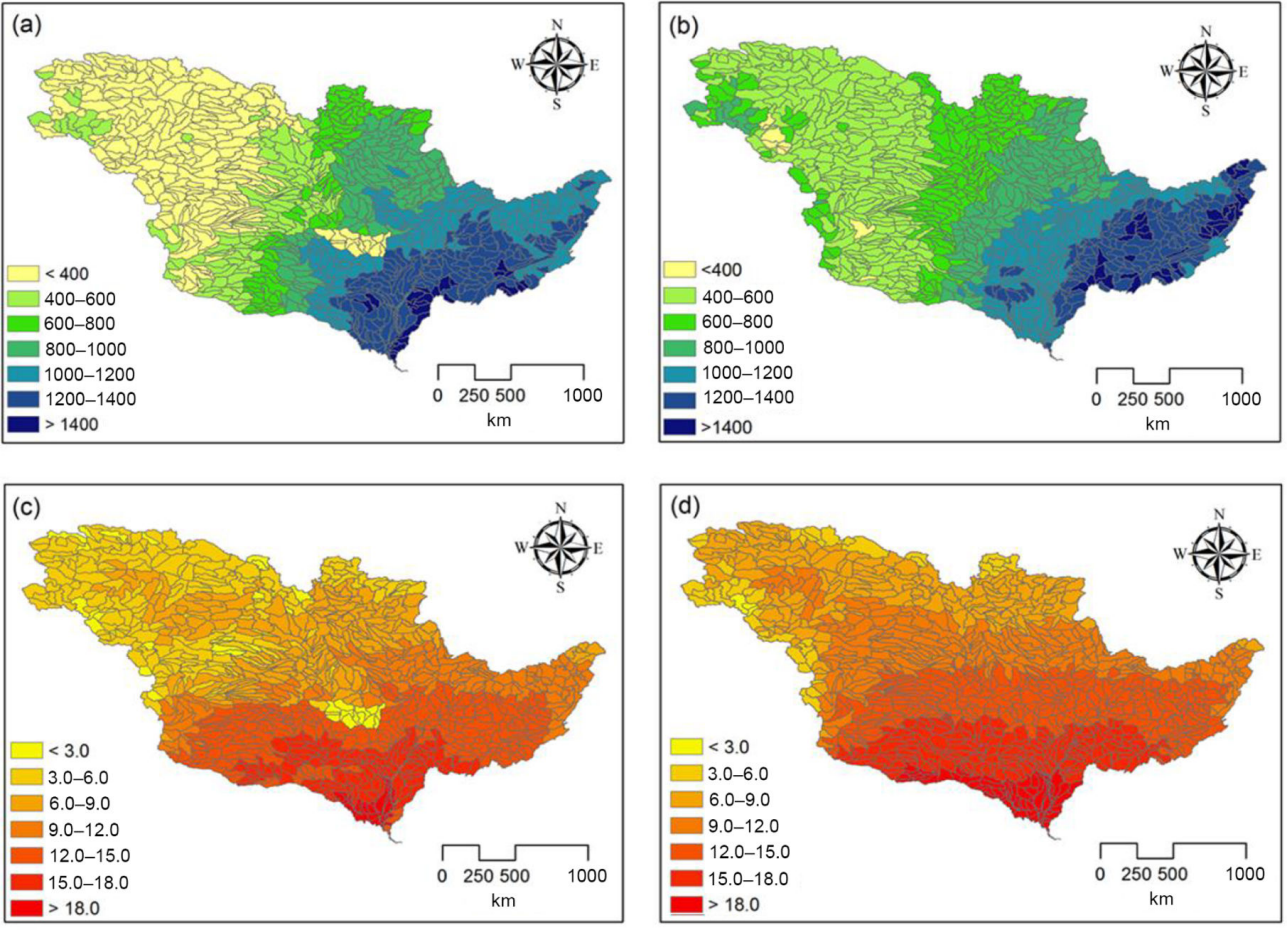
Comparison of average annual precipitation (mm) and temperature (°C) (2002–2010) between HAWQS and WRF climate at the HUC8 level: (**a**) HAWQS precipitation, (**b**) WRF precipitation, (**c**) HAWQS temperature, and (**d**) WRF temperature.

Since the only difference between HAWQS-SWAT and HAWQS-SWAT WRF simulations is the weather data, WRF-generated climate data were compared to HAWQS area-weighted NOAA-NCEI climate observations to understand how well the WRF model represents observed climate data in the study area. This is necessary because the FEST-C system was driven by process-based WRF weather simulations; thus, the IMS uses grid-based climate forcing by WRF because it is fully integrated with the air quality model CMAQ. WRF climate data of the 12 km by 12 km grid were aggregated to an 8-digit HUC level; this was also needed to run a HAWQS-SWAT WRF simulation, because HAWQS-SWAT requires one weather file for each subbasin. We compared the spatial distribution of average annual precipitation and air temperature from 2002–2010 (Fig. 3). This helped to understand the difference in streamflow simulations between HAWQS-SWAT and HAWQS-SWAT WRF and provided insights into IMS simulation results.

The trends in spatial distribution of precipitation across the MRB are similar between WRF simulations and NOAANCEI observations (Fig. 3a vs. Fig. 3b); the southeast of the MRB experienced higher annual precipitation than the northwest of the MRB. WRF systematically overestimated precipitation in the western Missouri River basin, however, and seemed to underestimate precipitation in the lower MRB (Fig. 3a vs. Fig. 3b). The trends in spatial distribution of temperature across the MRB are also similar between WRF simulations and NOAA-NCEI observations (Fig. 3c vs. Fig. 3d), but WRF seems to systematically overestimate temperature. Higher WRF precipitation would produce higher streamflow, but higher WRF temperature would result in higher evapotranspiration and, thus, lower streamflow.

**Figure 4.**
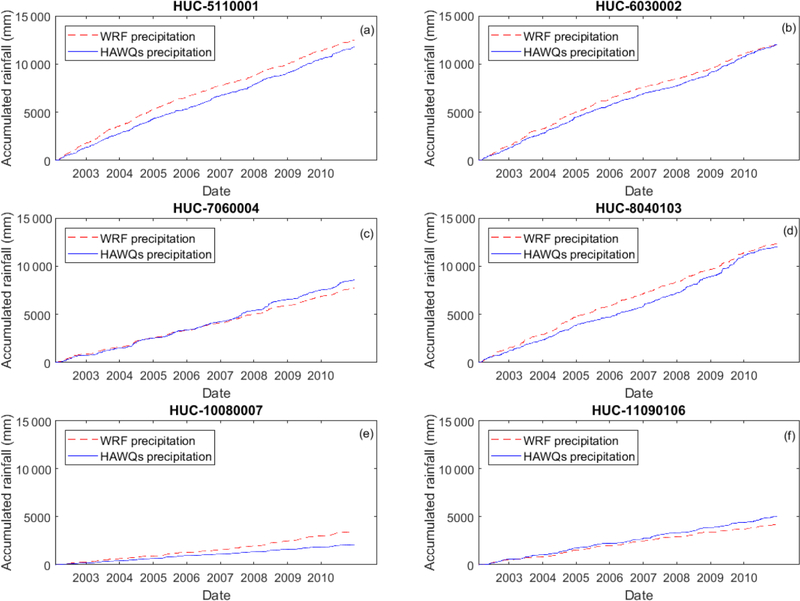
Rainfall accumulation curve comparison between NOAA-NCEI for HAWQS and WRF climate at randomly selected 8-digit HUCs:(**a**) Ohio River basin, (**b**) Tennessee River basin, (**c**) upper Mississippi River basin, (**d**) lower Mississippi River basin, (**e**) Missouri River basin, and (**f**) Arkansas Red–White River basin.

In addition to comparing the spatial distribution of average annual precipitation and temperature, we explored differences in daily precipitation patterns between NOAA-NCEI and WRF (Fig. 4). Daily precipitation accumulation curves from 2002 to 2010 at six randomly selected 8-digit HUCs (one from each 2-digit HUC) are presented in Fig. 4. The accumulative precipitation curve is similar between NOAA-NCEI observations and WRF simulations for all six 8-digit HUCs. WRF overestimated precipitation for the Ohio (Fig. 4a), lower Mississippi (Fig. 4d), and Missouri River basins (Fig. 4e) and underestimated precipitation for the upper Mississippi (Fig. 4c) and Arkansas Red–White River basins (Fig. 4f). For the Tennessee River basin, WRF overestimated precipitation from 2003 to 2008, but was close to the observations at the end of the comparison period. Overestimation of rainfall in the Missouri River basin is consistent with the spatial distribution presented in Fig. 4b. Since rainfall in the Ohio River basin is more than 10 000 mm for the 11-year accumulation, overestimation is small compared to total rainfall and would not substantially affect streamflow. In contrast, rainfall overestimation in the Missouri River basin (Fig. 4e) could introduce greater bias in hydrological modeling, because precipitation is less than 4000 mm for 11 years of accumulation. Although more comparisons between NOAA-NCEI and WRF need to be performed, the limited comparison shows that WRF can reproduce retrospective weather data.

### 3.2 Streamflow evaluation

**Figure 5.**
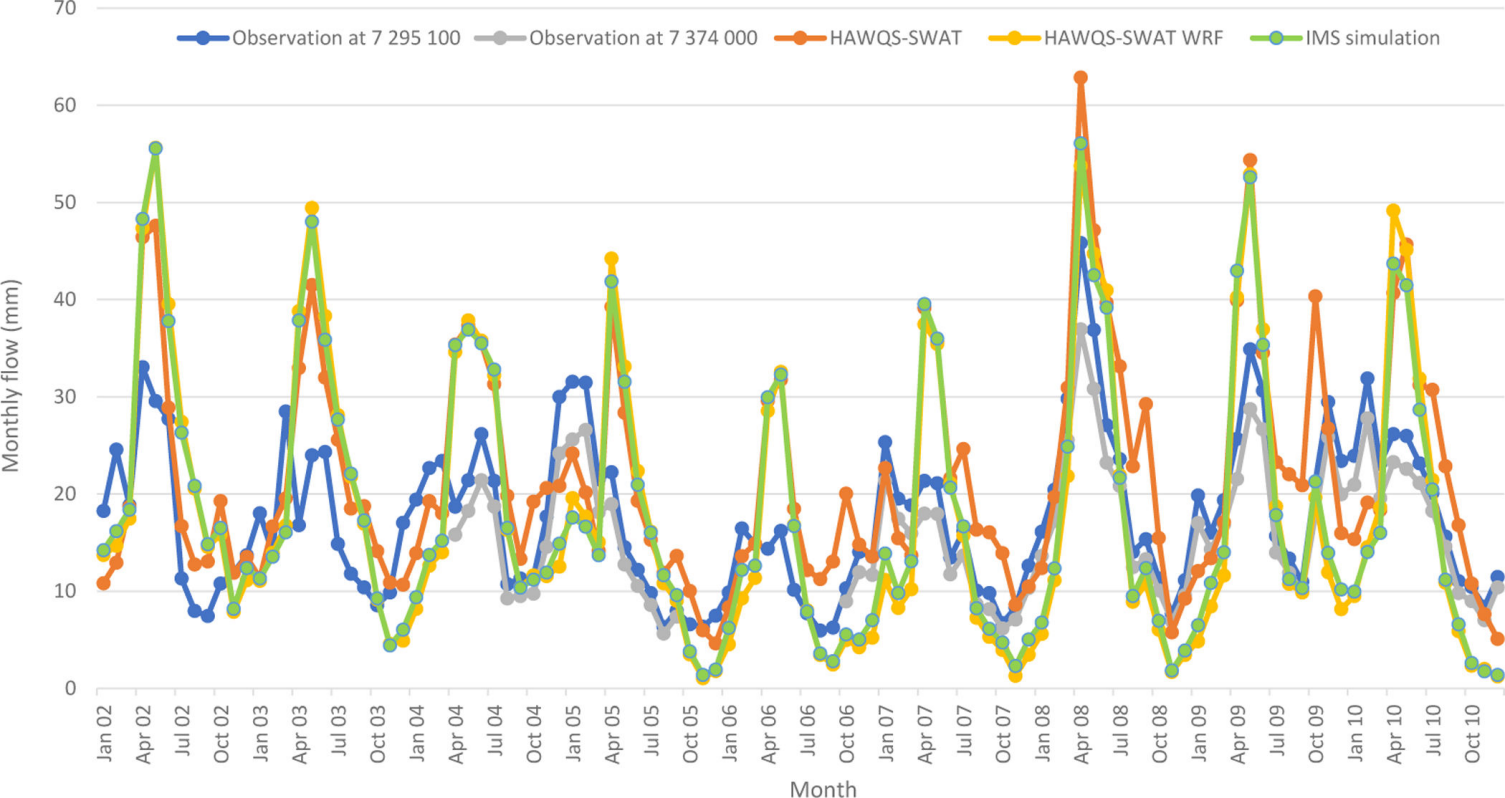
Monthly streamflow evaluation at USGS gauges.

Comparisons of simulated and observed monthly streamflow (USGS stations 07295100 at the Mississippi River at Tarbert Landing, MS; and 07374000 at Baton Rouge, LA) for the simulation period 2002 to 2010 are shown in Fig. 5. Generally, the simulated streamflow of HAWQS-SWAT followed the seasonal trends of the observed streamflow at 07295100, with an *R*^2^ of 0.52; *R*^2^ was not calculated for 07374000 due to the fact that 07374000 does not have complete data for the simulation period. Observations at 07374000 followed that of 07295100, with lower peaks (Fig. 5). The HAWQS-SWAT simulation overestimated streamflow, however, particularly for high flow months such as April in 2002, 2003, and 2008 and May in 2009 (Fig. 5). It also underestimated streamflow for the dry season such as December in 2009 and 2010 as well as January in 2006 and 2010. The average monthly flow observed at the USGS 07295100 was 17.6 mm and the simulated average monthly flow was 21.8 mm (Table 4).

Since the IMS simulation uses WRF-generated weather data, a simulation with WRF-generated weather data was also performed using the same inputs of HAWQS-SWAT, called HAWQS-SWAT WRF. The simulated streamflow of HAWQS-SWAT WRF followed the seasonal trends of the simulated streamflow of HAWQS-SWAT well, with an *R*^2^ of 0.83. The average monthly flow simulated by HAWQS-SWAT WRF is 18.0 mm, which is very close to the observed mean monthly flow (Table 4). The simulated streamflow of IMS is almost identical to the simulated flow of HAWQS-SWAT WRF, with an *R*^2^ of 0.99. The average monthly flow simulated by IMS is 18.2 mm (Table 4).

**Figure 6.**
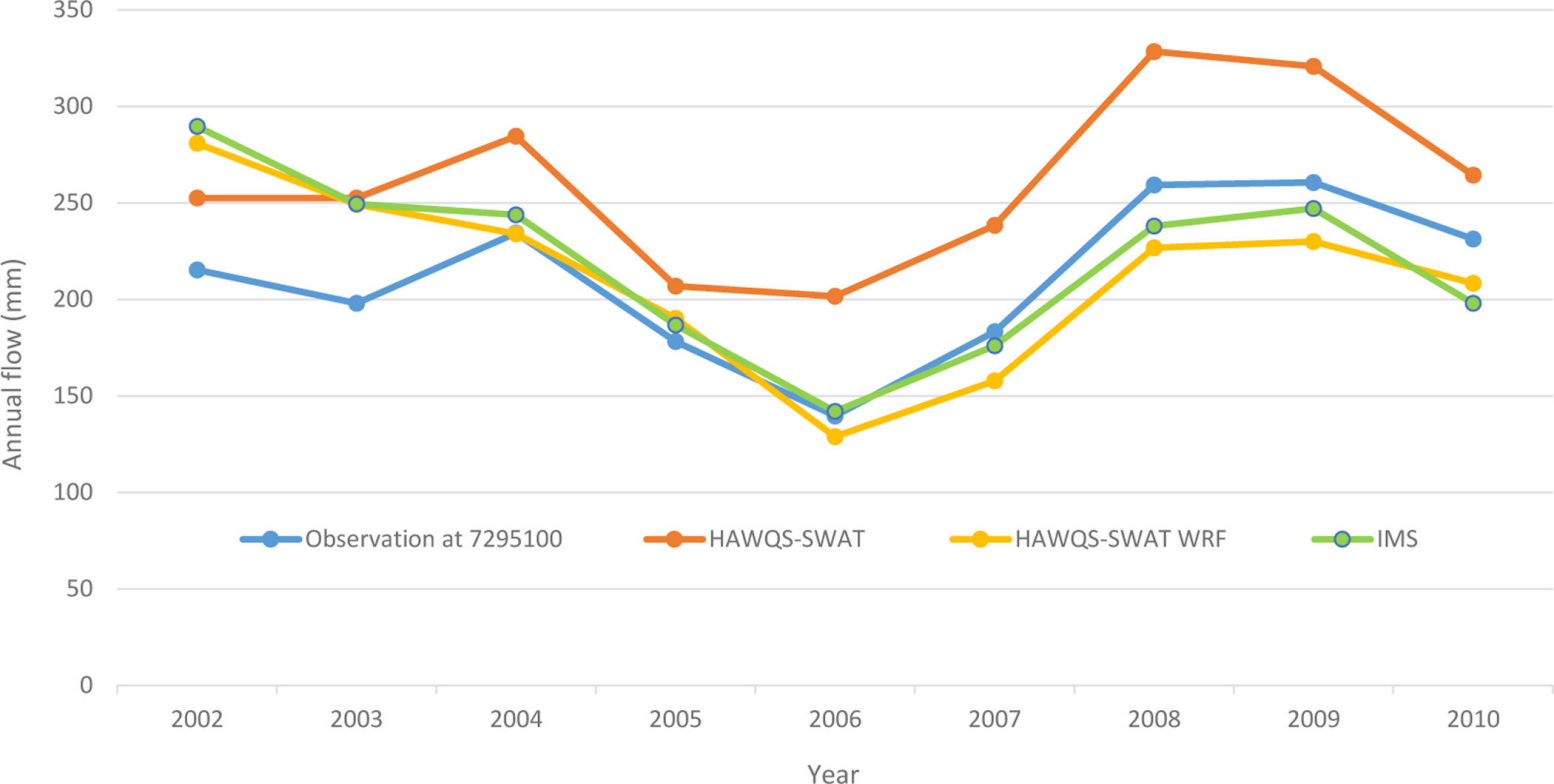
Annual streamflow evaluation at the USGS gauge.

The annual streamflow comparison of simulated and observed at the USGS station 07295100 at the Mississippi River at Tarbert is shown in Fig. 6; observed streamflow from the USGS station 07374000 at Baton Rouge, LA, was not shown because this station does not have all 9 years of data. Although the simulated streamflow of HAWQS-SWAT reflects the annual variation of the observed streamflow well, with an *R*^2^ of 0.90, it overestimated streamflow for all years of the simulation period (Fig. 6).

Runoff was possibly overestimated because SWAT underestimated groundwater recharge. As flows approach the GOM, water levels rise, resulting in higher seepage and groundwater recharge, a condition not well suited for SWAT modeling (Daggupati et al., 2016). Observations at 07374000 presented lower peaks (Fig. 5), which is consistent with this phenomenon. The lower groundwater recharge would result in lower baseflow, which also explains the underestimated monthly streamflow during the dry season (Fig. 5). Another possible reason for overestimation of the runoff is that water withdrawn for irrigation and other uses was not accounted for in the simulations.

In addition to the original calibration performed for 85 USGS stations where streamflow was available (HAWQS Quality Assurance Project Plan, an unpublished EPA document), further calibration is underway to expand on the initial calibration to improve HAWQS-SWAT’s performance. Calibration at such a scale, however, may be extremely difficult, as is demonstrated in the HAWQS QAPP and other studies (Daggupati et al., 2016; Scherer et al., 2015), due to the level of variability and uncertainty in streamflow. Determining how to calibrate the model effectively at such a scale, and with such high levels of variability and uncertainty (even conflicting results), would require a standalone study in the future; this is supported by other studies (Daggupati et al., 2016; Scherer et al., 2015).

The simulated annual streamflow of HAWQS-SWAT WRF is lower than the simulated annual streamflow of HAWQS-SWAT for all years but 2002. The average annual stream-flow simulated by HAWQS-SWAT WRF is 211.7 mm, which matched the observed mean annual streamflow of 211.1 (Table 4). The simulated annual streamflow of HAWQS-SWAT is 261.1. The simulated annual streamflow of IMS is very close to the simulated annual streamflow of HAWQS-SWAT WRF (Fig. 6). Higher WRF precipitation would produce higher streamflow, but higher WRF temperature would result in higher evapotranspiration and, thus, lower stream-flow; thus, lower simulated streamflow from HAWQS-SWAT WRF than from HAWQS-SWAT might be due to the combined effects of precipitation and temperature.

### 3.3 Dissolved N evaluation

**Figure 7.**
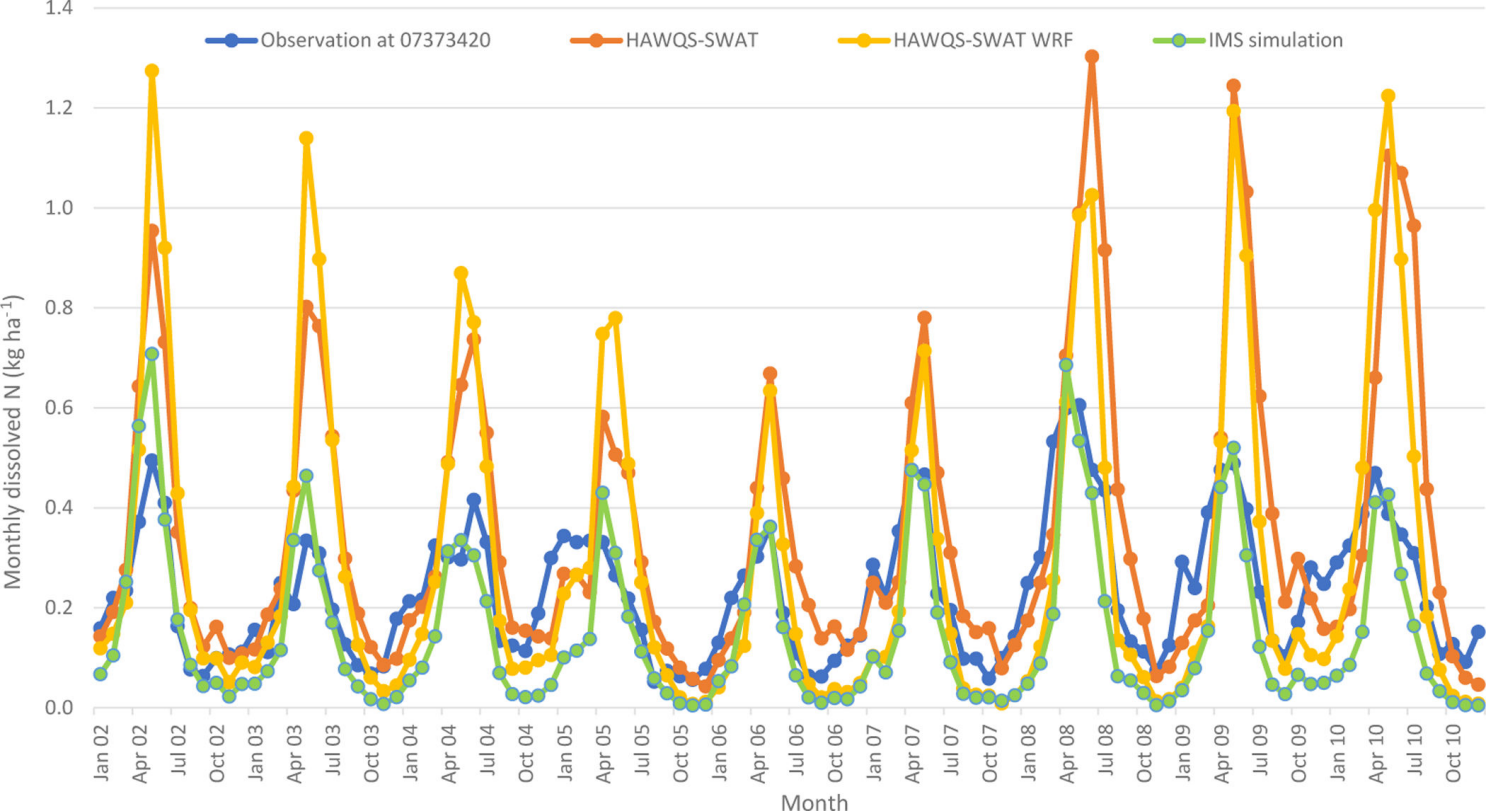
Monthly dissolved N evaluation at the total outlet of the MRB (USGS 07373420).

Comparisons of simulated and observed monthly dissolved N (USGS stations 07373420 at the Mississippi River near St. Francisville, LA) from 2002 to 2010 are shown in Fig. 7. Generally, the simulated dissolved N of HAWQS-SWAT followed the seasonal trends of the observed values, with an *R*^2^ of 0.53. The HAWQS-SWAT simulation overestimated dissolved N, as it did for streamflow (Fig. 3), particularly for spring and early summer months such as May in 2002, 2003, 2009, and 2010, as well as June in 2008 (Fig. 7). Fertilizer timing and amount impact N simulation as demonstrated in Yuan and Chiang (2015); fertilizer timing and amount in HAWQS were configured based on National Agricultural Statistics Service (NASS) county-level fertilizer sales information. It is very challenging to accurately capture fertilizer timing and amounts at such a large scale. The average monthly dissolved N estimated at the USGS 07373420 is0.23 kg ha^−1^ and the simulated amount by HAWQS-SWAT is 0.35 kg ha^−1^ (Table 4).

**Figure 8.**
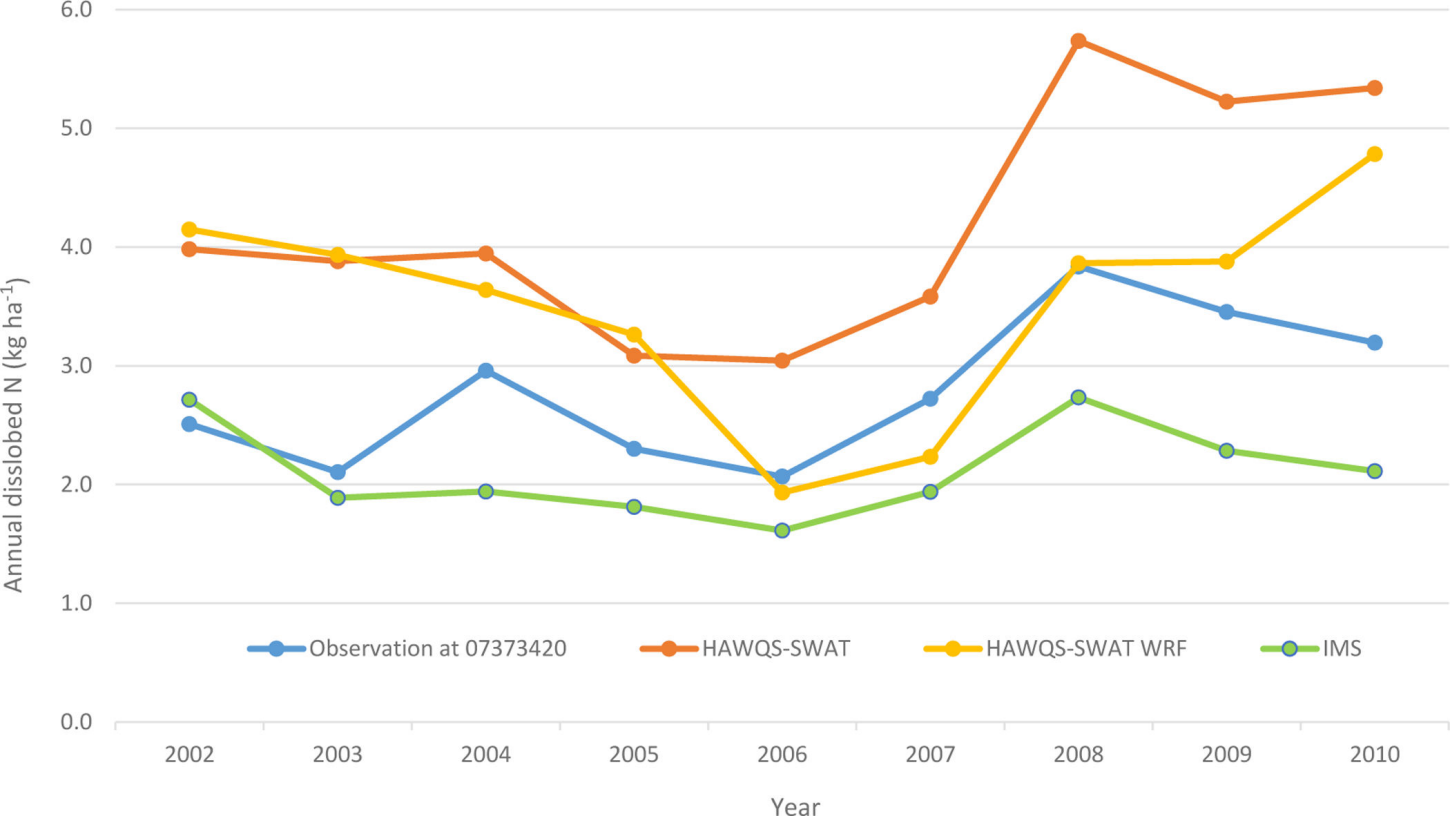
Annual dissolved N evaluation at the total outlet of the MRB (USGS 07373420).

The annual comparison between HAWQS-SWAT-simulated and observed dissolved N at the main outlet of the MRB (USGS 07373420) from 2002 to 2010 presents the same trends as the monthly results (Fig. 8). Model simulations of dissolved N correspond well to USGS estimations, with an *R*^2^ of 0.81. HAWQS-SWAT overestimated the dissolved N for all years during the simulation period, however (Fig. 8). Several potential factors could result in higher simulated dissolved N. First, higher runoff estimation could cause higher dissolved N results. In addition, fertilizer timing and amounts (as discussed above) could cause discrepancies. For the streamflow simulation, model calibration at such a scale with so much variability and uncertainty would be a daunting task. Evaluation of model simulations on nutrients has not been offered by the HAWQS developers. Finally, other studies (Chu et al., 2004; Grunwald and Qi, 2006; Hu et al., 2007; Yuan and Chiang, 2015) have demonstrated the disadvantage of SWAT models in simulating dissolved N, particularly in representing the impact of in-field processes on dissolved N.

The simulated dissolved N of HAWQS-SWAT WRF followed the seasonal trends of the simulated dissolved N of HAWQS-SWAT well, with an *R*^2^ of 0.85, although it results in overestimations and underestimations of dissolved N over the simulation period (Fig. 7). The average monthly dissolved N simulated by HAWQS-SWAT WRF is lower than HAWQS-SWAT (0.29 kg ha^−1^ vs. 0.35 kg ha^−1^) (Table 4). The lower simulated streamflow by HAWQS-SWAT WRF may result in lower simulated dissolved N. The simulated dissolved N of IMS is lower than the simulated dissolved N of HAWQS-SWAT WRF, but followed the seasonal trends of the HAWQS-SWAT WRF simulations, with an *R*^2^ of 0.78. The simulated dissolved N of IMS compared well to USGS estimations (*R*^2^ of 0.67), especially for the peaks, as shown in Fig. 7. Since EPIC was configured to simulate fertilization based on the plant demand using a computed N stress level during simulation, this results in higher fertilizer use efficiency and lower runoff loss compared with the fertilization information used by HAWQS-SWAT, which is from the USDA NASS county-level fertilizer sales information. Overall, the amount of fertilizer used in agricultural land in EPIC was 8 % lower than what was used in HAWQS-SWAT for the entire simulation period. The IMS, based on EPIC for agricultural land, simulates a wider variety of crop species and realistically represents crop growth and plant–nutrient relationships. Second, EPIC parameterizations have been selected to capture regional-scale crop production patterns, representative of a much finer scale of farm production practices. Finally, the IMS characterizes land–atmosphere N exchange in much greater detail. The IMS therefore demon strated greater advantages in simulating N processes than any previous work.

In summary, the IMS model was able to reflect seasonal variation of streamflow and dissolved N at USGS gauges, regardless of the complexity of the model, and variability and uncertainty of the watershed at such a large scale. For this proof of concept demonstration, model calibration was not performed. Model calibration at a scale with such variability and uncertainty is extremely difficult and offers the potential for a study in the future.

The IMS model integrated the previously developed FEST-C system with the SWAT model. The FEST-C system, driven by process-based WRF weather simulations, includes atmospheric N additions to agricultural cropland and agricultural cropland contributions to ammonia emissions. The IMS can assess impacts from meteorology, atmospheric N deposition, and agricultural management practices on water quality in large river basins.

## 4 Conclusions

The IMS is unique in its integration of climate, air deposition, landscape, and watershed processes (WRF/CMAQ/EPIC/SWAT), as well as its inclusion of detailed field-scale biogeochemistry on regional to national-scale simulations. It is an improvement of the existing FEST-C (CMAQ/WRF/EPIC) modeling system because stream and channel processes can be simulated after integrating the most commonly used watershed model, SWAT. On the other hand, IMS also improved SWAT simulation results, because it incorporates more field-scale biogeochemical processes by using EPIC in the FEST-C system for agricultural land simulations. Preliminary application of the IMS on MRB showed that simulation results are comparable to USGS observations (streamflow) and estimations (dissolvedN), particularly on dissolved N (annual simulated dissolved N of 2.1 kg ha^−1^ vs. USGS estimation of 2.8 kg ha^−1^).

## 5 Future work

Future work includes more evaluation of the model including baseflow, sediment, and organic N, using it to investigate additional potential sources of N from the watershed in a dynamic way and assessing the impact of CAA amendment regulations and land use and land management changes on N fate and transport in large river basins such as the MRB under alternative environmental scenarios. This marks a significant step forward toward a more complete systems-level framework for N assessment.

## Figures and Tables

**Table 1.** EPIC daily output variables converted to SWAT point source input.

EPIC output variable name	EPIC variable description	SWAT point source variable names	SWAT point source variable description	Conversion from EPIC to SWAT
Q/QDRN/SSF	Surface flow, tile drainage, and subsurface flow (mm)	FLODAY	Contribution to stream flow for the day (m^3^)	FLODAY = (Q+QDRN+SSF) × area
MUSL	Sediment loss (kg ha^−1^)	SEDDAY	Sediment loading to reach for the day (metric tons)	SEDDAY = (MUSL) × area × delivery ratio
YON	N loss with sediment (kg ha^−1^)	ORGNDAY	Organic N loading to reach for the day (kg N)	ORGNDAY = (YON) × area × delivery ratio
YP	P loss with sediment (kg ha^−1^)	ORGPDAY	Organic P loading to reach for the day (kg P)	ORGPDAY = (YP) × area × delivery ratio
QNO3/DRNN/SSFN	N loss in surface runoff, tile drainage, and subsurface flow (kg ha^−1^)	NO3DAY	NO_3_ loading to reach for the day (kg N)	N03DAY = (QNO3+DRNN+SSFN) × area
QAP/SSFP	Ploss in surface and subsurface flow (kg ha^−1^)	MINPDAY	Mineral P loading to reach for the day (kg P)	MINPDAY = (QAP+SSFP) × area

Area refers to HAWQS-SWAT agricultural land.

**Table 2.** N deposition variables used by SWAT and EPIC.

	SWAT		EPIC
Variable index	Variable name	Variable index	Variable name
1	Atmospheric wet deposition of ammonium (mg L^−1^) for the entire watershed	1	Daily total wet oxidized N (g ha^−1^)
2	Atmospheric wet deposition of nitrate (mg L^−1^) for the entire watershed	2	Daily total wet reduced N (g ha^−1^)
3	Atmospheric dry deposition of ammonium (kg ha^−1^ day^−1^) for the entire watershed	3	Daily total dry oxidized N (g ha^−1^)
4	Atmospheric dry deposition of nitrate (kg ha^−1^ day^−1^) for the entire watershed	4	Daily total dry reduced N (g ha^−1^)
		5	Daily total wet organic N (g ha^−1^)
		6	Daily total dry organic N (g ha^−1^)

**Table 3.** USGS monitoring stations close to the outlet of the MRB; size of the drainage area; and the time period for available discharge, sediment, and nitrogen data.

USGS monitoring station number	USGS monitoring station location	Watershed drainage area (km^2^)	Discharge		Nitrogen (nitrate plus nitrite)	
			Start	End	Start	End
07295100	Mississippi River at Tarbert Landing, MS	2913480	Jan 1930	Present	NA	
07373420	Mississippi River near St. Francisville, LA	2914516	No continuous flow monitoring		Oct 1943	Present
07374000	Mississippi River at Baton Rouge, LA	2915837	Apr 2004	Sep 2005	Dec 2011	Jan 2016
			Oct 2006	Apr 2016		

**Table 4.** Model evaluation for monthly and annual streamflow (mm) and dissolved N (kg ha^−1^) for the simulation period 2002–2010.

Constituents	Observation at USGS 7295100	Estimation at USGS 07373420	SWAT-HAWQS	SWAT-HAWQS WRF	IMS
Mean monthly streamflow (mm)	17.6		21.8	18.0	18.2
Mean annual streamflow (mm)	211.1		261.1	211.7	218.9
Mean monthly dissolved N (kg ha^−1^)		0.23	0.35	0.29	0.18
Mean annual dissolved N (kg ha^−1^)		2.8	4.2	3.5	2.1

## Appendix A: Abbreviations

CAA: Clear Air Act
CMAQ: Community Multiscale Air Quality model
EPIC: Environmental Policy Integrated Climate model
FEST-C: Fertilizer Emission Scenario Tool for the CMAQ model
HAWQS: Hydrologic and Water Quality System
MRB: Mississippi River basin
NASS: National Agricultural Statistics Service
NH_3_: ammonia
NH^+^_4_: ammonium
NO^−^_3_: nitrate
NOAA: National Oceanic and Atmospheric Administration
NCEI: National Centers for Environmental Information
NSTC: National Science and Technology Council
SWAT: Soil and Water Assessment Tool
USEPA: US Environmental Protection Agency
WRF: Weather Research and Forecasting Model
